# Incidence of and Mortality From Amyotrophic Lateral Sclerosis in National Football League Athletes

**DOI:** 10.1001/jamanetworkopen.2021.38801

**Published:** 2021-12-15

**Authors:** Daniel H. Daneshvar, Jesse Mez, Michael L. Alosco, Zachary H. Baucom, Ian Mahar, Christine M. Baugh, Jhaqueline P. Valle, Jennifer Weuve, Sabrina Paganoni, Robert C. Cantu, Ross D. Zafonte, Robert A. Stern, Thor D. Stein, Yorghos Tripodis, Christopher J. Nowinski, Ann C. McKee

**Affiliations:** 1 Department of Physical Medicine and Rehabilitation, Harvard Medical School, Boston, Massachusetts; 2 Department of Physical Medicine and Rehabilitation, Massachusetts General Hospital, Boston; 3 Department of Physical Medicine and Rehabilitation, Spaulding Rehabilitation Hospital, Boston, Massachusetts; 4 Boston University Alzheimer’s Disease and CTE Centers, Boston University School of Medicine, Boston, Massachusetts; 5 Department of Neurology, Boston University School of Medicine, Boston, Massachusetts; 6 Framingham Heart Study, Boston University School of Medicine, Boston, Massachusetts; 7 Department of Biostatistics, Boston University School of Public Health, Boston, Massachusetts; 8 Center for Bioethics and Humanities, University of Colorado Denver Anschutz Medical Campus, Aurora; 9 Division of General Internal Medicine, University of Colorado School of Medicine, Aurora; 10 Public Health Institute, California Department of Public Health, Richmond; 11 Department of Epidemiology, Boston University School of Public Health, Boston, Massachusetts; 12 Sean M Healey & AMG Center for ALS, Department of Neurology, Massachusetts General Hospital, Boston; 13 Department of Neurosurgery, Boston University School of Medicine, Boston, Massachusetts; 14 Department of Neurosurgery, Emerson Hospital, Concord, Massachusetts; 15 Concussion Legacy Foundation, Boston, Massachusetts; 16 Department of Physical Medicine and Rehabilitation, Brigham and Women’s Hospital, Boston, Massachusetts; 17 Department of Anatomy & Neurobiology, Boston University School of Medicine, Boston, Massachusetts; 18 Veterans Affairs Boston Healthcare System, Department of Veterans Affairs, Boston, Massachusetts; 19 Department of Veterans Affairs Medical Center, Bedford, Massachusetts; 20 Department of Pathology and Laboratory Medicine, Boston University School of Medicine, Boston, Massachusetts

## Abstract

**Importance:**

Amyotrophic lateral sclerosis (ALS) is a fatal neurodegenerative disease; understanding ALS risk factors is a critical public health issue.

**Objectives:**

To evaluate the incidence of and mortality from ALS in National Football League (NFL) athletes and to describe characteristics associated with ALS within this cohort.

**Design, Setting, and Participants:**

This population-based cohort study included all 19 423 NFL athletes who debuted between 1960 and 2019 and played 1 or more professional game. It was conducted between October 3, 2020, and July 19, 2021.

**Exposure:**

Participation in the NFL, including playing 1 or more professional games.

**Main Outcomes and Measures:**

Cases of ALS and death information were identified based on public records from NFL statistics aggregators, news reports, obituaries, and National Death Index results. The standardized incidence ratio and the standardized mortality ratio were calculated based on data acquired from surveillance studies of ALS accounting for age, sex, and race. Secondary analyses examined the association of body mass index, NFL career duration, race, birth location, and markers of fame, using a nested case-control design, matching athletes with ALS to athletes without ALS, by NFL debut year.

**Results:**

A total of 19 423 male former and current NFL players (age range, 23-78 years) were included in this cohort study and were followed up for a cumulative 493 168 years (mean [SD] follow-up, 30.6 [13.7] years). Thirty-eight players received a diagnosis of ALS, and 28 died during the study time frame, representing a significantly higher incidence of ALS diagnosis (standardized incidence ratio, 3.59; 95% CI, 2.58-4.93) and mortality (standardized mortality ratio, 3.94; 95% CI, 2.62-5.69) among NFL players compared with the US male population, adjusting for age and race. Among NFL athletes, nested-case-control analyses found that those who received a diagnosis of ALS had significantly longer careers (mean [SD] duration, 7.0 [3.9] years) than athletes without ALS (mean [SD] duration, 4.5 [3.6] years; odds ratio, 1.2; 95% CI, 1.1-1.3). There were no differences in ALS status based on proxies of NFL fame, body mass index, position played, birth location, or race.

**Conclusions and Relevance:**

The age-, sex-, and race-adjusted incidence of and mortality from ALS among all NFL players who debuted between 1960 and 2019 were nearly 4 times as high as those of the general population. Athletes with a diagnosis of ALS had longer NFL careers than those without ALS, suggesting an association between NFL duration of play and ALS. The identification of these risk factors for ALS helps to inform the study of pathophysiological mechanisms responsible for this fatal neurodegenerative disease.

## Key Points

**Question:**

What are the incidence and mortality rates of amyotrophic lateral sclerosis (ALS) in athletes who played in the National Football League (NFL)?

**Findings:**

In this cohort study of all 19 423 NFL athletes who debuted between 1960 and 2019 and played at least 1 professional game, the athletes had nearly 4 times higher incidence and mortality rates of ALS compared with the US male population, adjusting for age and race. Among these athletes, those with a diagnosis of ALS had played football for approximately 2.5 years longer than those without ALS.

**Meaning:**

This study’s findings suggest that factors associated with NFL participation may increase the incidence of and mortality from ALS.

## Teaser

This cohort study evaluates the incidence of and mortality from amyotrophic lateral sclerosis (ALS) in National Football League (NFL) athletes from 1960 to 2019 and describes characteristics associated with ALS within this cohort.

## Introduction

Amyotrophic lateral sclerosis (ALS) is a motor neuron disease characterized by loss of upper and lower motor neurons, often leading to rapidly progressing disability and death.^1^ The cause of this neurodegenerative process is not yet understood, with 90% of ALS cases considered sporadic.^1^ The incidence of ALS in the United States is 1.5 to 2.2 per 100 000 but varies markedly depending on age, sex, and race.^2,3,4,5,6,7,8^ The incidence of ALS generally increases with age and peaks during the seventh decade of life.^2,3,4,5^ Men have a higher incidence of ALS (1.7-2.6 per 100 000) than women (1.1-1.5 per 100 000),^2,3,4,5,6,7^ and White individuals (1.7-2.5 per 100 000) have a higher incidence than Black individuals (0.7-1.5 per 100 000).^2,3,6,7,8^ Because ALS is a fatal disease with a typically rapid course, its incidence rate largely approximates mortality.^9^ Given these substantial differences by age, sex, and race, adjusting for these variables is critical, especially in cohorts with unique characteristics such as elite athletes, as athletes often share characteristics that may be associated with ALS risk. Ideally, investigations of ALS risk in athletes would compare ALS incidence and mortality with nonathletes who otherwise have the same baseline ALS risks. Some studies have addressed this challenge of exchangeability of baseline risks by comparing ALS risk among elite athletes of 1 sport with ALS risk among elite athletes of another sport.^10^

Because ALS is fatal, understanding ALS risk factors is a critical public health issue both to improve knowledge of disease pathogenesis and to provide potential interventions to minimize these risks. Several putative risk factors associated with sporadic cases have been proposed, including repetitive head impacts (RHIs) and traumatic brain injury (TBI).^11,12,13,14^ Previous work has sought to quantify the association between ALS and TBI by studying ALS risk in individuals with high exposure to RHIs (ie, elite contact and collision sports athletes).^10,15,16^ Lehman et al^15^ examined mortality in the 3439 athletes who played at least 5 years in the National Football League (NFL) from 1959 through 1988, and found that there was an ALS age- and race-standardized mortality ratio of 4.04 (95% CI, 1.48-8.79), using US male mortality rates as the standard. Nguyen et al^10^ also examined mortality rates in a subset of these same NFL athletes, this time comparing them with 2708 former Major League Baseball players who played at least 5 seasons at the professional level from 1959 through 1988. They reported a race-adjusted ALS hazard ratio of 3.10 (95% CI, 0.84-11.38). Because the mean NFL career length is 3.3 years,^17^ restricting previous analyses to athletes who played for at least 5 years meant that only a subset of the most elite NFL players with greatest exposure was studied. In addition, athletes with shorter professional careers may have had early health consequences related to their athletic exposure, and/or different disease susceptibility compared with those with longer careers. Examining ALS risk in a more comprehensive group of athletes who participated in the NFL is necessary to better understand both the risk of ALS generally, as well as any possible association between duration of NFL play and ALS risk. In addition, because previous studies have relied on death certificates alone, the incidence of ALS in NFL players has not been previously reported, to our knowledge.

Other factors, which may be associated with NFL athletes, have been implicated in risk of ALS. Specifically, previous work has found that individuals with a higher body mass index (BMI; calculated as weight in kilograms divided by height in meters squared) have a lower risk of ALS 15 to 50 years later.^18^ In addition, environmental toxins are a proposed risk factor for ALS.^19^ Strenuous physical activity has also been implicated in the risk of ALS.^20,21,22^

We sought to clarify the association between ALS diagnosis and NFL participation by determining ALS incidence and mortality relative to the general population, and by examining the association between years of NFL exposure and ALS. We hypothesized that ALS incidence and mortality would be increased in NFL athletes compared with the general population, adjusting for age, sex, and race. To further characterize factors associated with ALS diagnosis, a nested case-control study matched by year of NFL debut examined differences based on duration of play, position, location of birth, and BMI at NFL debut. We hypothesized that NFL athletes with ALS would have significantly more years of NFL exposure, but no differences in position, BMI at debut, or location at birth, compared with NFL athletes without ALS. To assess the extent of selection bias associated with the link between publicity surrounding the announcement of ALS diagnosis and athlete fame, the nested case-control study also examined NFL Pro Bowl status and appearances, as well as NFL Hall of Fame status. We hypothesized that there would be no differences in markers of fame between NFL athletes with ALS and those without ALS.

## Methods

### Study Population

Information about all athletes drafted into the NFL since the league’s founding in 1920 is maintained in a comprehensive database by Hidden Game Sports/24-7 Baseball LLC, where it is licensed for use by major organizations including ESPN, Pro-Football Reference, SportRadar US, Stats Perform, and SportsTicker, and has been used for research purposes^23,24,25^ (see eMethods in the Supplement for additional details). Athletes were excluded from the study if they were not drafted from 1960 through 2019 or did not play in at least 1 regular season game professionally. The study was approved by the Boston University Medical Center institutional review board. This study followed the Strengthening the Reporting of Observational Studies in Epidemiology (STROBE) reporting guideline.

For the nested case-control study, a cumulative incidence sampling design was used to match 5 NFL athletes from the cohort without ALS to each athlete with ALS, based on debut year. Specifically, NFL athletes without ALS were randomly selected from all athletes who debuted that year within the cohort. The study was conducted between October 3, 2020, and July 19, 2021.

### Outcomes

Age at ALS diagnosis and death were identified based on public news reports (Google News) and obituaries (Legacy.com) to identify items containing the terms *NFL* and *ALS* through April 30, 2021. Flagged items were individually reviewed to identify public disclosures of ALS diagnoses in these NFL athletes. Age at death was obtained from the Hidden Game Sports/24-7 Baseball LLC database. Diagnoses of ALS at death were matched to National Death Index (NDI) records; NDI-Plus metrics were used to ensure matches between databases, with only class 3 or higher matches considered true matches. Athletes were included in the study from their date of NFL debut until ALS diagnosis and/or death.

### Statistical Analysis

Standardized incidence ratios and Fisher exact test 95% CIs were calculated from the expected incidence in the cohort. The expected incidence was determined using ALS incidence estimates from the largest US study of ALS incidence by age, sex, and race.^2^ In brief, crude incidence rates were calculated using the number of male patients with ALS with dates of initial diagnosis occurring from January 1, 2009, to December 31, 2011, along with age- and race-specific estimates. These rates were extrapolated for the entire study period. To ensure enough cases within each subgroup to generate an estimated incidence, we created age groups (20-49, 50-64, and ≥65 years) and classified race as Asian or Other Pacific Islander, Black, White, and other (races not conforming to Asian or Other Pacific Islander, Black, or White were classified as “other”) (eTable 1 in the Supplement). Denominator data came from the California Department of Finance Population Estimates.^26^ Standardized mortality ratio and Fisher exact test 95% CIs were calculated using the expected ALS mortality in the cohort, adjusted for age (in 5-year categories) and race, which was determined using the Centers for Disease Control and Prevention National Institute for Occupational Safety and Health Life Table Analysis System, version 4.5.^27^ Analyses used US male mortality rates with the neurodegenerative causes of death rate file (eTable 2 in the Supplement). These Life Table Analysis System data only provide mortality data for individuals classified as “White” and “non-White”; non-White was used as an imperfect proxy for Black for the purpose of mortality analyses alone.

For the nested case-control study, between-group differences were examined by conditional logistic regression to determine odds ratios with 95% CIs for each outcome. Primary playing position was determined, as well as grouping by nonspeed positions (offensive and defensive linemen) and speed positions (all other positions), to mirror previous analyses (eMethods in the Supplement).^15^ To account for multiple comparisons, false discovery rate–corrected *P* values for each category were calculated using the Benjamini-Hochberg procedure, with a significance threshold of .05.

In addition to the nested case-control study, logistic regression of the entire cohort was completed to confirm the association between duration of play and ALS status, adjusting for BMI, debut year, and race. All statistical analyses were conducted with IBM SPSS Statistics, version 20.0 (IBM Corp) and R Statistical Software, version 3.6.1 (R Group for Statistical Computing).

## Results

A total of 19 423 male former and current NFL players (age range, 23-78 years) were included in the cohort and followed up for a cumulative 493 168 years (mean [SD] follow-up, 30.6 [13.7] years). A total of 55 professional football players with ALS diagnoses were identified. Eight of these athletes were reported as NFL athletes but did not play a game in the NFL, and 9 debuted before 1960; thus, 38 NFL athletes with ALS were part of the cohort (Figure). Characteristics of NFL athletes with ALS are described in Table 1 and eFigure in the Supplement. These athletes received a diagnosis of ALS at a mean (SD) age of 51.0 (13.8) years. A total of 28 athletes died during the study window; 24 of the 28 were identified in a database of NDI-Plus results, with 23 including a clear underlying cause of death (the remaining case was described as “ill-defined and unknown cause of mortality”). Of these 23 athletes, 22 were confirmed to have ALS as an underlying entity or cause of death (the cause of death for the remaining case was described as “myocardial infarction”). The decedents were diagnosed at a mean (SD) age of 52.5 (13.8) years and lived for a mean (SD) of 3.5 (2.6) years with ALS. The 10 living athletes received a diagnosis of ALS at a mean (SD) age of 46.1 (12.6) years, and have been alive with ALS for a mean (SD) of 6.1 (5.7) years. There was no significant difference between living and deceased athletes in age at ALS diagnosis (*P* = .23) or years between diagnosis and death or end of study (*P* = .06), although the mean values are quite different, and our study may have been inadequately powered to detect differences owing to the low number of athletes with ALS. The standardized incidence ratio was 3.59 (95% CI, 2.58-4.93) and the standardized mortality ratio was 3.94 (95%CI, 2.62-5.69; Table 2). Although both the standardized incidence ratio and standardized mortality ratio were lower for White athletes compared with Black athletes (standardized incidence ratio: 3.50 [95% CI, 2.24-5.21] vs 3.63 [95% CI, 1.93-6.21]; and standardized mortality ratio: 3.61 [95% CI, 2.14-5.71] vs 4.72 [95% CI, 2.26-8.67]), these differences were not statistically significant.

**Figure.**
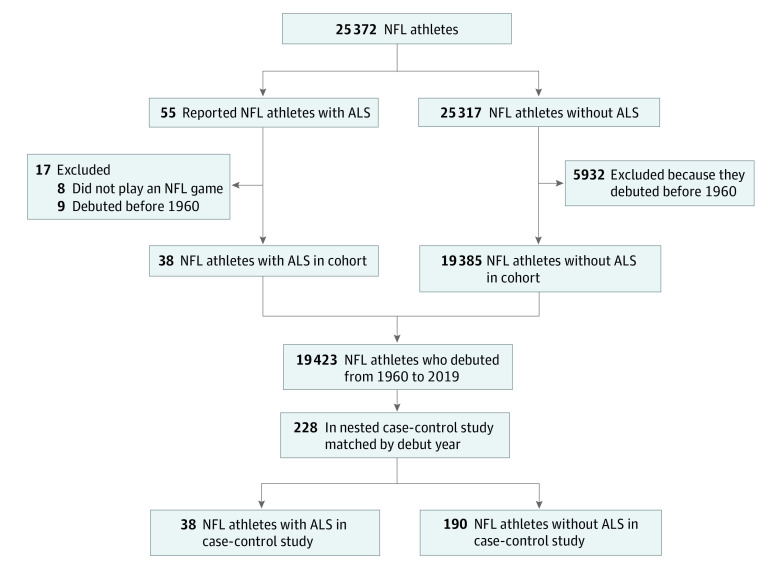
Study Inclusion and Exclusion Criteria for National Football League (NFL) Cohort and Nested Case-Control Analyses ALS indicates amyotrophic lateral sclerosis.

**Table 1.** Characteristics of NFL Athletes With ALS Who Debuted Between 1960 and 2019

Decade of debut	No. of ALS cases	No. of athletes by race	Position	Time in NFL, mean (SD), y	Age at entering NFL, mean (SD), y	Age at ALS diagnosis, mean (SD), y	Time before death, mean (SD), y [No. of athletes]	Time between diagnosis and end of study, mean (SD), y [No. of athletes]^a^
1960s	15	1 Black and 14 White players	2 DB, 3 DL, 1 LB, 4 OL, 3 QB, 1 RB, 1 WR	6.3 (4.1)	23.1 (1.5)	60.4 (12.8)	3.2 (2.8) [15]	NA
1970s	5	1 Black and 4 White players	1 DL, 1 LB, 1 OL, 1 RB, 1 WR	8.8 (3.1)	22.4 (0.5)	55.8 (10.9)	3.0 (2.2) [4]	4.0 (0.0) [1]
1980s	13	10 Black and 3 White players	4 DB, 2 DL, 3 LB, 2 OL, 2 RB	6.6 (4.8)	22.6 (1.0)	45.3 (10.1)	3.0 (1.8) [7]	4.8 (7.2) [6]
1990s	3	2 Black and 1 White player	1 DB, 1 LB, 1 RB	7.3 (0.6)	24.3 (2.3)	37.0 (4.6)	8.0 (2.8) [2]	13.0 (0.0) [1]
2000s	2	2 White players	1 DB, 1 LB	6.5 (0.7)	23.0 (0.0)	32.0 (2.8)	NA	7.5 (2.1) [2]
2010s	NA	NA	NA	NA	NA	NA	NA	NA
Total	38	14 Black and 24 White players	8 DB, 6 DL, 7 LB, 7 OL, 3 QB, 5 RB, 2 WR	6.8 (3.9)	22.9 (1.3)	51.0 (13.8)	3.5 (2.6)	6.1 (5.7)

Abbreviations: ALS, amyotrophic lateral sclerosis; DB, defensive backs; DL, defensive linemen; LB, linebackers; NA, not applicable; NFL, National Football League; OL, offensive linemen; QB, quarterbacks; RB, running backs; WR, wide receivers.

^a^ Study window ended on April 30, 2021.

**Table 2.** Amyotrophic Lateral Sclerosis Incidence and Mortality in NFL Athletes

NFL players	Standardized incidence ratio (95% CI)	Standardized mortality ratio (95% CI)
Overall	3.59 (2.58-4.93)	3.94 (2.62-5.69)
Race		
Black	3.63 (1.93-6.21)	4.72 (2.26-8.67)
White	3.50 (2.24-5.21)	3.61 (2.14-5.71)

Abbreviation: NFL, National Football League.

Athletes with ALS played in the NFL for a mean (SD) of 7.0 (3.9) years compared with 4.5 (3.6) years for matched athletes without ALS (odds ratio, 1.2; 95% CI, 1.1-1.3; *P* ≤ .001). There were no differences between athletes with ALS and matched athletes without ALS in debut BMI, race, Pro Bowl status, number of NFL Pro Bowl appearances, NFL Hall of Fame status, or position (Table 3), as well as location of birth (eTable 3 in the Supplement). Proxies of football fame were associated with duration of play (eTable 4 in the Supplement). Logistic regression over the entire cohort demonstrated a significant association between ALS and duration of NFL play as well as debut year, but not race or BMI (eTable 5 in the Supplement).

**Table 3.** Differences Between NFL Athletes With ALS and Matched NFL Athletes Without ALS

Characteristic	Athletes with ALS (n = 38)	Matched athletes without ALS (n = 190)	Odds ratio (95% CI)^a^
Potential ALS risk factors			
Years of NFL play, mean (SD)	7.0 (3.9)	4.5 (3.6)	1.2 (1.1 to 1.3)^b^
BMI at debut, mean (SD)	29.3 (3.2)	29.0 (3.4)	1.0 (0.9 to 1.1)
Black race, No. (%)	14 (36.8)	103 (54.2)	2.0 (1.0 to 4.2)
Markers of fame			
Ever in an NFL Pro Bowl, No. (%)	5 (13.2)	18 (9.5)	1.5 (0.5 to 4.2)
NFL Pro Bowl appearances, mean (SD)	0.18 (0.51)	0.14 (0.49)	1.2 (0.6 to 2.3)
NFL Hall of Fame, No. (%)	0	3 (1.6)	Not applicable
Position, No. (%)^c^			
Nonspeed vs speed	12 (31.6)	53 (27.9)	0.33 (−0.80 to 1.46)
All positions, individual, No. (%)			
Defensive back	7 (18.4)	30 (15.8)	*P* = .50^d^
Defensive line	6 (15.8)	22 (11.6)	
Linebacker	7 (18.4)	23 (12.1)	
Offensive line	6 (15.8)	31 (16.3)	
Quarterback	3 (7.9)	6 (3.2)	
Running back	7 (18.4)	40 (21.1)	
Special teams	0	1 (0.5)	
Tight end	1 (2.6)	14 (7.4)	
Wide receiver	1 (2.6)	22 (11.6)	

Abbreviations: ALS, amyotrophic lateral sclerosis; BMI, body mass index (calculated as weight in kilograms divided by height in meters squared); NFL, National Football League.

^a^ Difference between athletes with ALS and matched athletes without ALS. The 95% CI from the χ^2^ test was used for analyses of categorical variables, with the Fisher exact test used when expected values were less than 5, or from 2-sample *t* test for analyses of continuous variables.

^b^ Significance based on false discovery rate–corrected *P* < .05.

^c^ Analyses examined differences among all 9 positions, as well as grouped by nonspeed (offensive and defensive linemen) and speed positions (all other positions).

^d^ *P* value was determined using the Fisher exact test and was provided given no odds ratio for more than 2 × 2 tables.

## Discussion

To our knowledge, this study represents the largest investigation of ALS risk in NFL athletes. These findings indicate that athletes who played in the NFL have a nearly 4 times greater rate of developing, and dying from, ALS.

Previous analyses have reported similar increased rates of ALS in former NFL athletes.^10,15^ However, these studies examined only mortality, and only in athletes who played from 1959 through 1988 for at least 5 years at the professional level. The present study is the first, to our knowledge, to report ALS incidence in NFL athletes. In addition, by including all athletes who debuted from 1960 through 2019 and who played in at least 1 NFL game, the present study expands the number of athletes studied by nearly 6-fold. By including a wider range of NFL exposures, the present study is also the first, to our knowledge, to show that greater duration of NFL play is associated with increased ALS rates among NFL athletes.

There are 2 potential explanations for the latter finding: (1) either duration of NFL play serves as a proxy for an exposure that increases risk of ALS or (2) there were additional athletes with a diagnosis of ALS who also had shorter NFL careers but were not identified for the present study. Although it is certainly likely that announcements pertaining to more famous NFL athletes generate more news coverage, all NFL athletes garner news coverage at different stages throughout their playing career; even athletes with relatively unknown professional careers were often standout football stars at the local, high school, and/or collegiate level. In addition, there were no differences in proxies of football fame, including NFL draft position, NFL Pro Bowl status, number of NFL Pro Bowl appearances, or NFL Hall of Fame status, between NFL athletes who received a diagnosis of ALS and those who did not. The ALS mortality rate reported in this cohort is also similar to that reported by prior studies.^10,15^ Furthermore, the 55 athletes with ALS identified in the present study are largely consistent with the 59 individuals with ALS granted monetary awards as part of the NFL concussion settlement.^28^

In addition, the game of football has changed substantially throughout the past 60 years. Several of these changes may be associatedwith ALS risk. For example, the racial composition of the NFL has changed from predominantly White athletes to predominantly Black athletes.^29^ Older athletes, therefore, are more likely than younger athletes to be White. Because both age and race have a nonlinear association with ALS risk, both these factors should be accounted for simultaneously when examining the role of NFL participation in the risk of ALS. Furthermore, style of play and how positions are used has changed substantially, which may have a further association with ALS risk. The NFL officially adopted plastic shell helmets in 1943, single-bar face masks in 1955, and double-bar face masks in 1962, with each resulting in significant changes to RHI and TBI risk.^30,31^ Statistically adjusting for these confounders may not adequately account for the intersection of these factors, whereas a case-control study matched for debut year better accounts for these interacting confounders.

Ultimately, this study provides additional evidence suggesting that NFL athletes are at increased risk of ALS and suggests that this risk may increase with more years of NFL exposure. There were no associations between ALS and location at birth, position played, or BMI, although the low number of athletes with ALS limits the power to detect differences. Although the present design is unable to identify the specific factor(s) responsible for an increased rate of ALS among NFL players, it is possible that RHIs or TBIs play a role. Previous studies have demonstrated a dose-response association between duration of football career and the neurodegenerative disease chronic traumatic encephalopathy (CTE).^32,33^ Pathologic evidence has suggested that CTE and ALS may be linked based on the TDP-43 (transactive response DNA-binding protein 43)–positive inclusions in many cases of CTE, especially marked in the subset of individuals with neuropathologically confirmed CTE and ALS.^13,25,34^ Furthermore, in a study of 155 veterans with ALS who donated their brain for research, 9 (5.8%) were found to have neuropathologic evidence of CTE, and those with ALS and CTE were more likely to have a history of TBI, bulbar-onset ALS symptoms, mood disorders, and behavioral disorders.^14^ Other factors that may explain the observed increase in risk of ALS, such as smoking, physical exertion, and athletic exposure to substances such as pesticides, are potentially relevant.^12,19,20,35,36^ However, studies comparing ALS rates in elite football players with those in elite baseball players have found an approximately 3-fold higher hazard ratio for the football players, which supports RHIs and/or TBIs as a more likely risk rather than physical exertion or pesticide exposure.^10^

### Limitations

There are several limitations to the present study. Both the retrospective nature of the study and identification of cases from public records could cause underrepresentation of the true incidence and mortality of ALS within this cohort.^37^ In addition, without access to clinical information, it is possible that some of these cases do not truly represent diagnosed ALS; however, this possibility is less likely given the secondary confirmation of 22 of the 23 decedents with a clear underlying cause of death in the NDI records, the short duration between diagnosis and death for most of these athletes, and the similar duration after diagnosis for decedents and those currently alive. Also owing to the lack of clinical information, the study could not assess the presence of clinical differences, or other risk factors, including family history, genetics, environmental toxin exposure, or lifestyle factors (eg, smoking). Data pertaining to RHI exposure and TBI history, both within and outside of football, were also unavailable. Amyotrophic lateral sclerosis diagnoses in NFL athletes were based on news reports, potentially resulting in variability in the capture of cases compared with medical record review, which is how the general population incidence of ALS was determined. However, given that ALS is a fatal disease, mortality largely approximates incidence.

In addition, ALS diagnoses in the general population were based on data from 2009 through 2011, compared with 1960 through 2021 in the NFL cohort; changes in ALS incidence over time may confound this comparison. Given the low number of ALS cases, the study was underpowered to detect smaller differences; null findings, particularly regarding location at birth and position, should be interpreted in this context. Furthermore, duration of play and position at the NFL level do not represent total football exposure; athletes start playing football at a range of ages,^38,39^ and this cumulative exposure likely plays a role in the risk of neurodegenerative disease.^32,40,41^ Finally, the total person-years that the cohort in this study was deemed at risk of ALS was based on date of NFL debut and date of death; the latter was determined from NFL statistics aggregators and may not have captured all deaths. However, this would bias toward a larger denominator, and thus an artificially lower calculated rate of ALS for the cohort.

## Conclusions

Amyotrophic lateral sclerosis is a fatal disease with a significant socioeconomic and public health burden. Identifying potential exposure-related risk factors that can be modified or reduced is important so that these risks can be minimized and eliminated. These findings from NFL football players may also provide insight into other groups that share specific exposures, such as individuals playing other contact sports and military veterans with RHI exposure,^14,42,43^ elite athletes with high physical activity,^20,21,22,35,44^ and gardeners with pesticide and fertilizer exposure.^19^ Further study is therefore warranted to clarify the causal mechanisms underlying the association between duration of professional football play and incidence of ALS.

## Notes

Supplement.
eMethods

eReferences
**eTable 1.** Crude Amyotrophic Lateral Sclerosis Incidence Rates per 100,000 Males **eTable 2.** Crude Amyotrophic Lateral Sclerosis Mortality Rates per 100,000 Males **eTable 3.** Differences in Location of Birth Between NFL Athletes With ALS and Matched NFL Athletes Without ALS **eTable 4.** Association Between Proxies of Football Fame and Duration of NFL Play **eTable 5.** Logistic Regression Examining Effect of Duration of Play on ALS Status **eFigure.** Number of NFL Athletes by Number of NFL Years Played
