# Supplementary material for: Incidence of and Mortality From Amyotrophic Lateral Sclerosis in National Football League Athletes
Source: JAMA Netw Open. 2021 Dec 15;4(12):e2138801. doi: 10.1001/jamanetworkopen.2021.38801 (PMC8674746; doi:10.1001/jamanetworkopen.2021.38801)
Supplement: Supplement. — eMethods eReferences eTable 1. Crude Amyotrophic Lateral Sclerosis Incidence Rates per 100,000 Males eTable 2. Crude Amyotrophic Lateral Sclerosis Mortality Rates per 100,000 Males eTable 3. Differences in Location of Birth Between NFL Athletes With ALS and Matched NFL Athletes Without ALS eTable 4. Association Between Proxies of Football Fame and Duration of NFL Play eTable 5. Logistic Regression Examining Effect of Duration of Play on ALS Status eFigure. Number of NFL Athletes by Number of NFL Years Played [file jamanetwopen-e2138801-s001.pdf]

Supplementary Online Content

Daneshvar DH, Mez J, Alosco ML, et al. Incidence of and mortality from amyotrophic lateral sclerosis in National Football League athletes. *JAMA Netw Open.* 2021;4(12):e2138801. doi:10.1001/jamanetworkopen.2021.38801

eMethods
eReferences
eTable 1. Crude Amyotrophic Lateral Sclerosis Incidence Rates per 100,000 Males
eTable 2. Crude Amyotrophic Lateral Sclerosis Mortality Rates per 100,000 Males
eTable 3. Differences in Location of Birth Between NFL Athletes With ALS and Matched NFL Athletes Without ALS
eTable 4. Association Between Proxies of Football Fame and Duration of NFL Play
eTable 5. Logistic Regression Examining Effect of Duration of Play on ALS Status
eFigure. Number of NFL Athletes by Number of NFL Years Played

This supplementary material has been provided by the authors to give readers additional information about their work.

© 2021 Daneshvar DH et al. *JAMA Network Open.*

eMethods

Study Population.

The Hidden Game Sports & 24-7 Baseball, L.L.C. database includes information pertaining to each athlete, including date of birth, date of death, location of birth (city, state/country), race, NFL games played, NFL draft position, height at NFL debut, weight at NFL debut, duration of NFL career, number of NFL Pro Bowl appearances, and NFL Hall of Fame status. Prior to the 1966 merger of the National Football League and American Football Leagues, many athletes were drafted in both leagues. For the purpose of the present study, athletes from both leagues were considered NFL players. The entire cohort was obtained from the comprehensive Hidden Game Sports database, which only considers athletes as NFL players if they played in at least one game. However, potential subjects with ALS were generated from news and obituary reports (the left pathway in Figure 1). Some news and obituary reports described athletes as NFL players with ALS, but they did not meet the specific criteria for inclusion in this cohort (i.e., play in at least one NFL game).

Position.

Positions were grouped into the following nine categories: Defensive back (including Cornerback, Safety, Halfback), Defense line (including Tackle, End, Nose Tackle), Linebacker, Offensive line (including Guard, Tackle, Center), Quarterback, Running back (including Halfback, Fullback), Special team (including Kicker and Punter), Tight end, and Wide receiver (including Split End and Flanker). For the 15 athletes with multiple positions across these categories, a single position was selected based on recent data from college football players; the position with the highest lifetime number of self-reported concussion was selected.[1,2] Analyses examined differences between all nine positions, as well as grouped by non-speed (offensive and defensive linemen) and speed positions (all other positions), to mirror previous analyses.[3]

© 2021 Daneshvar DH et al. *JAMA Network Open.*

eReferences

1. Baugh CM, Kiernan PT, Kroshus E, et al. Frequency of head-impact-related outcomes by position in NCAA division I collegiate football players. *J Neurotrauma*. 2015;32(5):314-326. doi:10.1089/neu.2014.3582

2. Daneshvar DH, Nowinski CJ, McKee A, Cantu RC. The Epidemiology of Sport-Related Concussion. *Clin Sports Med*. 2011;30(1):1-17. doi:10.1016/j.csm.2010.08.006

3. Lehman EJ, Hein MJ, Baron SL, Gersic CM. Neurodegenerative causes of death among retired National Football League players. *Neurology*. 2012;79(19):1970-1974. doi:10.1212/WNL.0b013e31826daf50

4. Valle J, Roberts E, Paulukonis S, Collins N, English P, Kaye W. Epidemiology and surveillance of amyotrophic lateral sclerosis in two large metropolitan areas in California. Amyotroph Lateral Scler Frontotemporal Degener. 2015;16(3-4):209-215. Medline:25822003 doi:10.3109/21678421.2015.1019516

5. State of California, Department of Finance. State and county population projections 2010-2060. Accessed April 27, 2021. https://www.dof.ca.gov/Forecasting/Demographics/Projections/

6. Centers for Disease Control and Prevention. Life table analysis system—rate file information. Accessed April 6, 2021. https://www.cdc.gov/niosh/ltas/rates.html

© 2021 Daneshvar DH et al. *JAMA Network Open.*

eTable 1. Crude Amyotrophic Lateral Sclerosis Incidence Rates per 100,000 Males

Age	Asian/Pacific Islander	Black	White	Other
20-49	0.53	1.00	1.46	0.29
50-64	1.60	1.65	4.45	0.73
65+	5.45	7.36	10.77	2.46

Crude incidence rate based on individuals living in Los Angeles and San Francisco metro areas diagnosed with amyotrophic lateral sclerosis between 1/1/2009 and 12/31/2011.[4] To ensure enough cases within each subgroup to generate an estimated incidence, we created age groups 20-49, 50-64, and 65+ years. Denominator data came from the California Department of Finance Population Estimates.[5]

© 2021 Daneshvar DH et al. *JAMA Network Open.*

eTable 2. Crude Amyotrophic Lateral Sclerosis Mortality Rates per 100,000 Males

Age	White	Non-White
15-19	0.01	0.00
20-24	0.02	0.03
25-29	0.05	0.04
30-34	0.14	0.12
35-39	0.34	0.21
40-44	0.71	0.49
45-49	1.27	0.96
50-54	2.18	1.34
55-59	3.58	2.01
60-64	5.42	3.15
65-69	7.80	3.78
70-74	9.77	4.66
75-79	11.07	5.14
80-84	10.45	5.10
85+	7.55	2.75

Data obtained from Centers for Disease Control National Institute for Occupational Safety and Health Life Table Analysis System.[6]

© 2021 Daneshvar DH et al. *JAMA Network Open.*

eTable 3. Differences in Location of Birth Between NFL Athletes With ALS and Matched NFL Athletes Without ALS

Birth Location (state or non-US country)	Athletes with ALS (n=38)	Matched athletes without ALS (n=190)	p-value
Alaska	0	1	0.68
Alabama	2	6	
Arkansas	0	2	
Arizona	0	1	
California	1	16	
Connecticut	0	1	
Washington D.C.	1	2	
Delaware	0	1	
Florida	1	11	
Georgia	1	4	
Iowa	1	2	
Illinois	1	8	
Indiana	0	4	
Kansas	0	2	
Kentucky	0	4	
Louisiana	4	8	
Maine	0	4	
Maryland	0	2	
Michigan	0	10	
Minnesota	0	3	
Missouri	0	4	
Mississippi	1	6	
North Carolina	3	5	
North Dakota	0	1	
Nebraska	1	2	

© 2021 Daneshvar DH et al. *JAMA Network Open.*

New Hampshire	0	1	
New Jersey	0	3	
New York	1	4	
Ohio	3	16	
Oklahoma	0	1	
Oregon	1	1	
Pennsylvania	2	8	
South Carolina	0	5	
Tennessee	0	3	
Texas	8	20	
Utah	0	1	
Virginia	2	4	
Washington	1	4	
Wisconsin	0	1	
West Virginia	0	1	
Canada	0	1	
England	1	1	
Germany	1	0	
Turkey	1	0	
Unknown	0	5	

Amyotrophic lateral sclerosis: ALS, NFL: National Football League

© 2021 Daneshvar DH et al. *JAMA Network Open.*

eTable 4. Association Between Proxies of Football Fame and Duration of NFL Play

Proxy	Mean (std)	p-value
Hall of fame	13.5 years (2.8)	<0.001
Not Hall of Fame	4.7 years (3.7)	
Pro Bowl Appearance	10.7 years (3.5)	<0.001
No Pro Bowl Appearances	4.2 years (3.2)	

NFL: National Football League

© 2021 Daneshvar DH et al. *JAMA Network Open.*

eTable 5. Logistic Regression Examining Effect of Duration of Play on ALS Status

Regression Term	Odds Ratio (95%CI)	p
Years of NFL play	1.1 (1.03, 1.17)	<0.001
Debut year	0.95 (0.93, 0.97)	<0.001
BMI	1.01 (0.92, 1.11)	0.72
Race: White	1.67 (0.97, 2.37)	0.15

ALS: amyotrophic lateral sclerosis; BMI: basal metabolic index; CI: confidence interval

Logistic regression adjusting for debut year, BMI, and race, across all NFL Athletes who debuted between 1960 through 2019.

© 2021 Daneshvar DH et al. *JAMA Network Open.*

eFigure. Number of NFL Athletes by Number of NFL Years Played

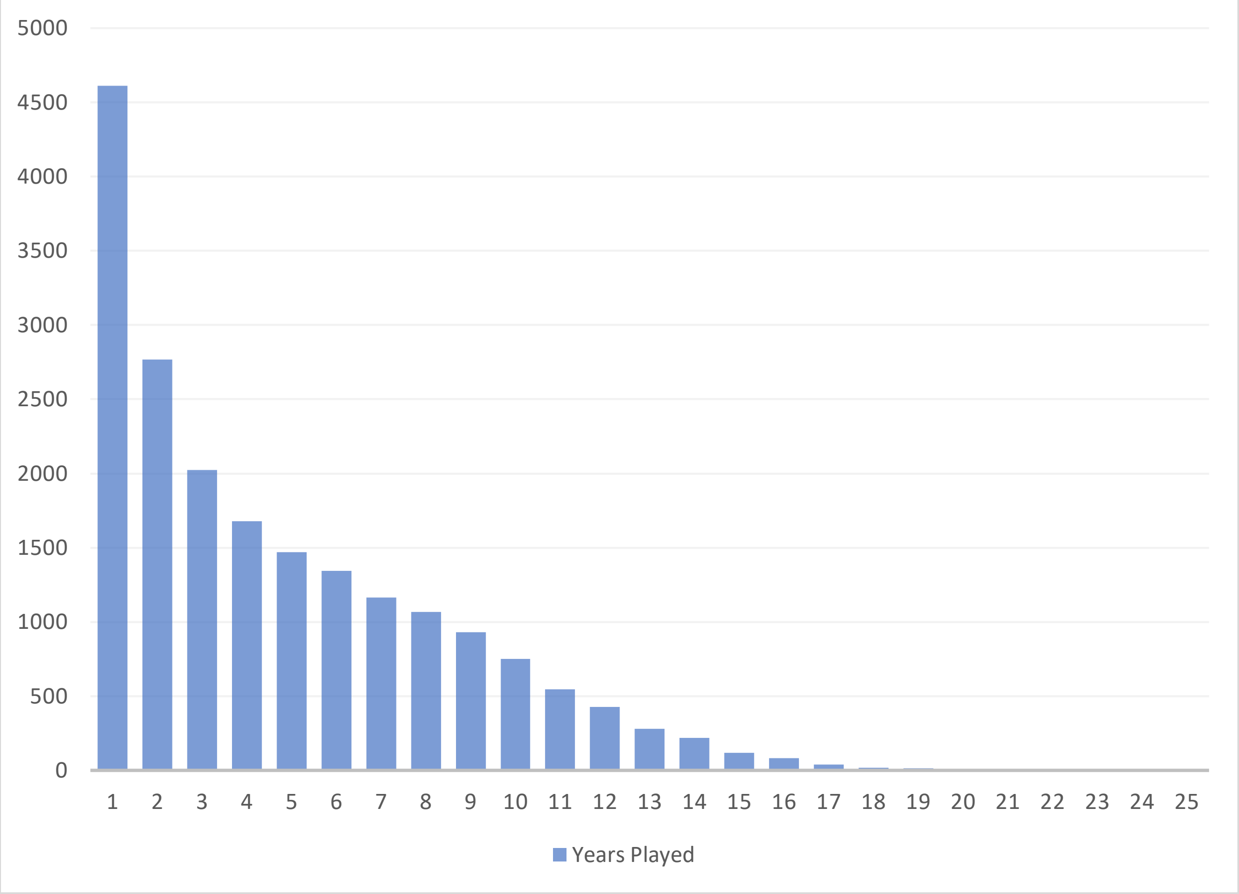

NFL: National Football League

© 2021 Daneshvar DH et al. *JAMA Network Open.*
